# Relationships between serum free fatty acid and pulse pressure amplification in overweight/obese men: insights from exercise training and dietary modification

**DOI:** 10.3164/jcbn.17-103

**Published:** 2018-03-30

**Authors:** Toru Yoshikawa, Asako Zempo-Miyaki, Hiroshi Kumagai, Kanae Myoenzono, Rina So, Takehiko Tsujimoto, Kiyoji Tanaka, Seiji Maeda

**Affiliations:** 1 Graduate School of Comprehensive Human Sciences, University of Tsukuba, 1-1-1 Tennoudai, Tsukuba, Ibaraki 305-8577, Japan; 2 Faculty of Health and Sport Science, Ryutsu Keizai University, 120 Ryugasaki, Ibaraki 301-8555, Japan; 3 Faculty of Health and Sport Science, Juntendo University, 1-1 Hirakagakuendai, Inzai, Chiba 270-1606, Japan; 4 Research Center for Overwork-Related Disorders, National Institute of Occupational Safety and Health, 6-21-1 Nagao, Tama-ku, Kawasaki, Kanagawa 214-8585, Japan; 5 Faculty of Human Sciences, Shimane University, 1060 Nishikawatsu, Matsue, Shimane 690-8504, Japan; 6 Faculty of Health and Sport Sciences, University of Tsukuba, 1-1-1 Tennoudai, Tsukuba, Ibaraki 305-8577, Japan

**Keywords:** physical activity, energy restriction, weight loss, non-esterified fatty acid, blood pressure

## Abstract

Pulse pressure amplification (i.e., the ratio of peripheral to central pulse pressure) is a strong predictor of cardiovascular events. Circulating free fatty acid, which is a major cause of insulin resistance, has been reported to favorably be associated with pulse pressure amplification in the arm (from the aorta to brachial artery). We hypothesized that this paradoxical relationship depended on an evaluating site of pulse pressure amplification and investigated whether serum free fatty acid level is related to pulse pressure amplification in the arm or trunk (from the aorta to femoral artery) in overweight/obese men. In a cross-sectional study, 85 men participated, and regression analyses revealed that serum free fatty acid level was significantly and independently associated with pulse pressure amplification in the arm but not the trunk. In a longitudinal study, 33 men completed a 12-week lifestyle intervention that involved both exercise training and dietary modification. The lifestyle intervention-induced change in serum free fatty acid level was significantly correlated to that in pulse pressure amplification in the arm but not the trunk. These results support our hypothesis and suggest that pulse pressure amplification should be measured in the trunk instead of the arm in overweight/obese men to simplify its interpretation.

## Introduction

Management of blood pressure is an essential part of reducing cardiovascular risk. Blood pressure is usually assessed at brachial artery in clinical practice, but several studies reported that noninvasively estimated aortic pressure is a better predictor of cardiovascular outcomes beyond brachial blood pressure.^(1–3)^ Recently, a decreased peripheral/central pressure ratio has been suggested as a stronger mechanical biomarker for predicting cardiovascular events than central or peripheral blood pressure alone.^(4,5)^ The pressure difference between proximal and distal arteries, which is called “pulse pressure amplification” (PPA) toward the periphery, is an important physiological function to protect the heart against after load and microcirculation from pulsatile pressure stress.^(6)^ PPA is commonly evaluated between the aorta and brachial artery (PPA in the arm) or femoral artery (PPA in the trunk).^(7)^ Although PPA is principally determined by reflections of propagated pressure waves,^(7)^ PPA in the arm does not consider the major reflection site (i.e., aortic and femoral bifurcations).^(8)^

Serum level of free fatty acid (FFA), the main cause of insulin resistance, has been reported to be favorably associated with PPA from aorta to brachial artery in general population.^(9)^ This paradoxical relationship makes the interpretation of PPA difficult. Tabara *et al.*^(9)^ speculated that the favorable relationship between serum FFA and PPA in the arm could be explained by a FFA-induced disappearance of stiffness gradient along the arterial tree, which decreases wave reflection from a resistant artery. Because a reflected pressure wave from the lower body determines peak blood pressure at the aorta and femoral artery but not the brachial artery,^(7)^ the relationship between serum FFA and PPA might depend on an evaluating site of PPA (i.e., trunk or arm). In addition, the association between serum FFA and PPA was observed by only cross-sectional study; therefore, it should be confirmed by a longitudinal study.

Thus, the purpose of the present study was to examine, in a cross-sectional and longitudinal (lifestyle intervention) design, whether serum FFA level is associated with PPA in the arm or trunk. We hypothesized that serum FFA level is cross-sectionally related to PPA in the arm, but not the trunk, and a lifestyle intervention–induced change in FFA is associated with that in PPA in the arm but not trunk. To highlight the role of FFA, this study enrolls overweight/obese adults who reportedly have a higher circulating FFA concentration compared to lean controls.^(10)^

## Methods

### Participants

Participants were recruited through local newspaper advertisements. The inclusion criteria were as follows: (a) men; (b) 30–64 years old; (c) body mass index (BMI) ≥25 kg/m^2^. The exclusion criteria were as follows: (a) presence or history of cardio/cerebrovascular disease (assessed via a medical history questionnaire). A total of 85 men were enrolled in a cross-sectional study, and subsequently 33 men participated in a 12-week lifestyle intervention. In accordance with the World Health Organization’s international classification, participants with a BMI of ≥25.0 kg/m^2^ were classified as overweight/obese. All procedures were reviewed and approved by the ethics committee of the University of Tsukuba and conformed to the principles of the Declaration of Helsinki. All participants gave written informed consent prior to participation.

### Measurements

Participants were instructed to avoid exercise for at least 24 h before the measurements. The participants fasted overnight for 10–12 h, which includes abstaining from the consumption of caffeine, alcohol, medication and smoking (only water was allowed). All measurements were conducted in a laboratory that was maintained at 24–26°C.

Anthropometric measurements were taken when the participants were barefoot and wearing only light clothing. Height was measured to the nearest 0.1 cm using a wall-mounted stadiometer (YG-200; Yagami, Nagoya, Japan). Body mass was measured to the nearest 0.1 kg on a calibrated digital scale (WB-150; Tanita, Tokyo, Japan). BMI was calculated as body mass divided by height squared (kg/m^2^). Waist circumference at the level of the umbilicus in a standing position was measured directly on the skin to the nearest 0.1 cm with flexible tape (in duplicate, then averaged).

Hemodynamic parameters were measured in the supine position after at least 20 min of resting. Brachial systolic and diastolic blood pressures were measured with oscillometric pressure sensor cuffs (form PWV/ABI; Colin, Komaki, Japan). The average of the two blood pressure records was used for data analysis. Pulse pressure was determined as the difference between systolic and diastolic blood pressure. Mean blood pressure was calculated as diastolic blood pressure plus 40% of brachial pulse pressure.^(11,12)^ Pressure pulse waveforms of the left common carotid artery and left common femoral artery were simultaneously obtained using two applanation tonometers (form PWV/ABI; Colin). The carotid waveforms were resampled from 1,200 Hz to 128 Hz with data acquisition and analysis software (AcqKnowledge 3.7.3; BIOPAC Systems, Goleta, CA) and were used to estimate aortic systolic blood pressure with a generalized transfer function (SphygmoCor 8.0; AtCor Medical, Sydney, Australia). Based on the physiological principal that mean and diastolic blood pressures remain almost unchanged from central elastic artery to peripheral muscular artery,^(7)^ the aortic and femoral pressure waveforms were calibrated by mean and diastolic blood pressures.^(13)^ Time to reflection was defined as round-trip travel time of the pressure waveform between the heart and the effective reflection site and was calculated as the time lag between initial upstroke of aortic pressure and the systolic inflection point. Ejection duration was determined as the time from initial upstroke of aortic pressure and the dicrotic notch of pressure. Heart rate was calculated from the cardiac cycle derived from aortic pressure waveforms. Pulse pressure amplification from the aorta to femoral (PPA_aorta__-femoral_) was calculated as the percentage ratio of femoral pulse pressure to aortic pulse pressure. Pulse pressure amplification from the aorta to brachial (PPA_aorta__-brachial_) was calculated as the percentage ratio of brachial pulse pressure to aortic pulse pressure. Pulse wave velocity from carotid to femoral (PWV_carotid-femoral_) was determined as 80% of the direct distance between carotid and femoral divided by the transit time (foot-to-foot method).^(14)^ Pulse wave velocity from femoral to ankle (PWV_femoral-ankle_) was determined as the direct distance between femoral and ankle divided by the transit time.

Blood samples were taken from the antecubital vein when fasting to measure the plasma levels of glucose and the serum levels of high-density lipoprotein (HDL) cholesterol, low-density lipoprotein (LDL) cholesterol, triglycerides, free fatty acid and insulin which were determined using standard enzymatic techniques. The homeostasis model assessment for insulin resistance (HOMA-IR) was calculated from the fasting insulin and glucose levels using the following equation: HOMA-IR = Fasting glucose (mg/dl) × Fasting insulin (mU/L)/405. The following conversion factors were used to convert conventional units to SI units: HDL and LDL cholesterol from mg/dl to mmol/L, multiply by 0.0259; triglycerides from mg/dl to mmol/L, multiply by 0.0113; free fatty acid from mEq/L to µmol/L, multiply by 1,000; glucose from mg/dl to mmol/L, multiply by 0.0555; insulin from µU/ml to pmol/L, multiply by 6.945.

### Lifestyle intervention

The participants took part in a 12-week lifestyle intervention, which consisted of both exercise training program (90 min per session, three days a week) and dietary modification program (90 min per session, a day a week) as described in our previous reports with minor modifications.^(15–18)^ The exercise training program included a 15–20 min warm-up session followed by an approximate 40–60 min walking and/or jogging session and concluded with a 15–20 min cool-down session. The exercise intensity was maintained at approximately 60–85% of the participant’s age-predicted maximal heart rate using a portable heart rate monitor (Polar RS400^TM^; Polar Electro Oy, Kempele, Finland). Participants were also encouraged to undertake further unsupervised physical activity while at home. The dietary modification program included both group lectures and individual counseling sessions by trained staff, and the participants were instructed on switching to a nutritionally well-balanced hypocaloric diet. The diet program was based on the four-food-group point method and aimed to limit the total energy intake of participants up to 1,680 kcal (21 points; 1 point = 80 kcal) per day allowing a self-selection of diet: 240 kcal (3 points) per day from food group I (e.g., dairy products and eggs);^(19)^ 480 kcal (6 points) per day from food group II (e.g., meat, fish and beans); 240 kcal (3 points) per day from food group III (e.g., vegetables, fruits and seaweeds); and 720 kcal (9 points) per day from food group IV (e.g., grains, nuts, snacks, oil, sugar, beverages and alcohol). During the program, the participants were asked to record their body weight and details of all food consumed in their daily diary and calculate the food points step by step. The trained staff added compliments and advice in each participant’s diary during the lecture and provided short counseling and encouragement after the lecture.

### Statistical analyses

Statistical analyses were performed using SPSS Statistics 24.0 for Windows (IBM, Tokyo, Japan). The Shapiro–Wilk test was used to evaluate the normality of distributions. The determinants of PPA were explored by linear regression models in the cross sectional study. The differences between before and after the lifestyle intervention were assessed by paired *t* tests or Wilcoxon signed-rank tests, as appropriate. The relationships between lifestyle intervention–induced changes in both serum FFA level and PPA in the trunk or arm were assessed with Pearson’s product moment coefficient (*r*). In all tests, a two-tailed *p*<0.05 was accepted as statistically significant. Data are expressed as the mean ± SD or frequency counts and percentages (%).

## Results

In the cross-sectional study, subjects’ characteristics are presented in Table 1. There were 26 (31%) patients using medications for hypertension, 13 (15%) patients using medications for dyslipidemia, (11%) patients using medications for hyperglycemia, and 21 (25%) current smokers. Table 2 summarizes the determinants of PPA_aorta-femoral_ and PPA_aorta-brachial_. PPA_aorta-femoral_ were significantly and independently related to time to reflection, ejection duration, and PWV_carotid-femoral_. PPA_aorta-brachial_ were significantly and independently associated with LDL cholesterol, FFA, ejection duration, and PWV_carotid-femoral_.

**Fig. 1:**
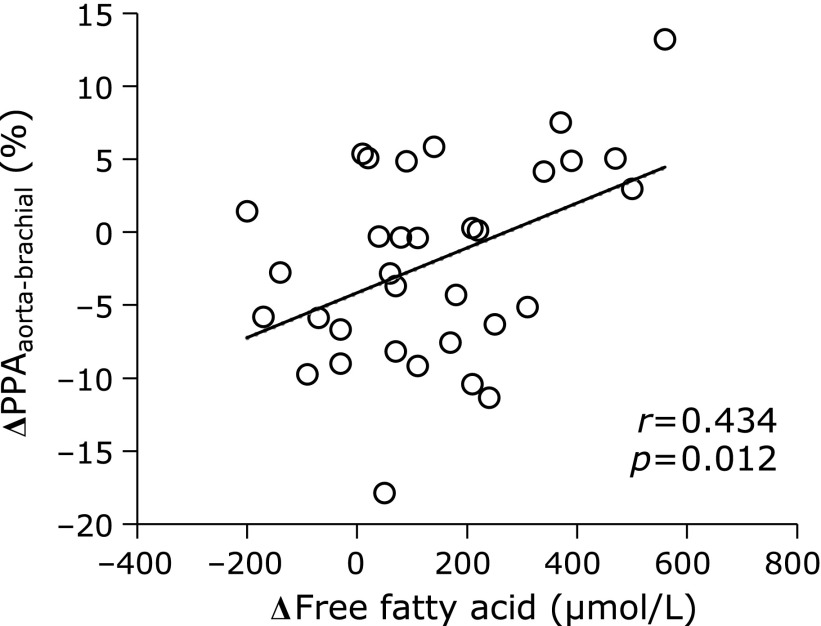
The relationship between changes in both PPA_aorta-brachial_ and serum free fatty acid level after the 12-week lifestyle intervention. PPA, pulse pressure amplification.

**Fig. 2:**
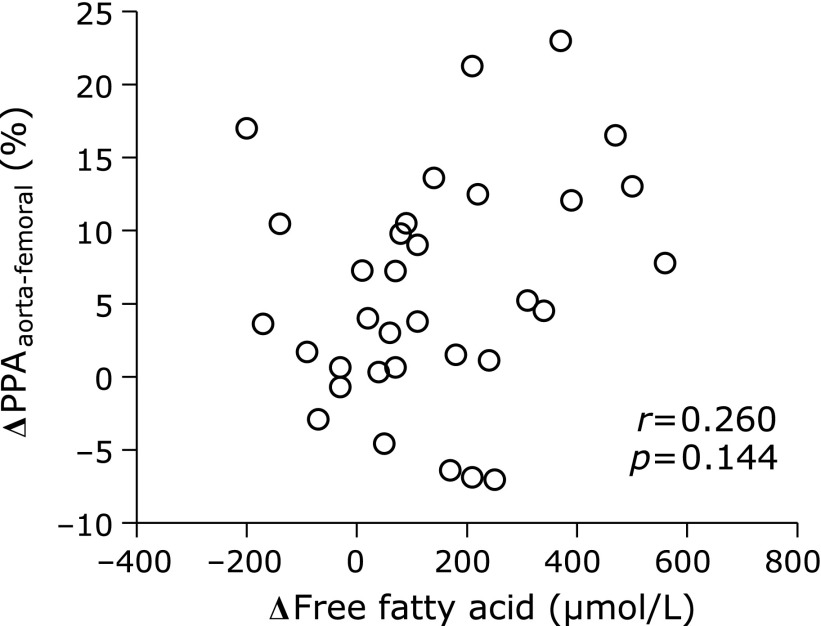
The relationship between changes in both PPA_aorta-femoral_ and serum free fatty acid level after the 12-week lifestyle intervention. PPA, pulse pressure amplification.

In the longitudinal study, participants’ characteristics before and after the 12-week lifestyle intervention are shown in Table 3. There were 10 (30%) patients using medications for hypertension, 7 (21%) patients using medications for dyslipidemia, no patients using medications for hyperglycemia and 6 (18%) current smokers. After the intervention, PPA_aorta-femoral_ significantly increased, while PPA_aorta-brachial_ remained unchanged. Serum FFA levels increased significantly after the intervention. The change in serum FFA level after the intervention was significantly associated with the change in PPA_aorta-brachial_ (Fig. 1) but not with PPA_aorta-femoral_ (Fig. 2).

## Discussion

This study aimed to examine the relationship between serum FFA level and PPA in overweight/obese men, and we focused on the different properties of PPA in the trunk (from the aorta to femoral) and arm (from the aorta to brachial). In the cross-sectional study, serum FFA level was significantly associated with PPA in the arm but not the trunk. In the longitudinal study (a 12-week exercise training and dietary modification), lifestyle intervention–induced change in serum FFA level was significantly correlated to that in PPA in the arm but not the trunk. These results support our hypothesis that the relationship between serum FFA and PPA depends on an evaluating site of PPA.

Serum FFA and subsequent insulin resistance have been reported to be paradoxically associated with PPA in the arm.^(20,21)^ In conformity with the previous reports, the regression model of the present study showed that serum FFA level was an independent factor that favorably influences PPA in the arm. FFA is a major causative factor of insulin resistance,^(22)^ which suppresses insulin-stimulated endothelial nitric oxide release, and compensatory hyperinsulinemia augments both endothelin-1 production and sympathetic nerves activity.^(23)^ Indeed, acute elevation of circulating FFA level attenuates the endothelium-dependent vasorelaxation.^(24)^ Through these mechanisms, FFA is suggested to diminish stiffness gradient from central elastic artery to peripheral muscular artery, limiting partial reflection of pressure wave from periphery.^(9)^ Limited wave reflection from the lower body can decrease systolic blood pressure at the aorta but not brachial artery,^(7)^ which leads to decreased aortic pressure relative to brachial pressure and increased PPA in the arm. However, present results showed that the influence of circulating FFA on PPA in the arm was independent of both central and peripheral PWV, indicating other pathways that FFA favorably relates to PPA in the arm.

We found for the first time that there was no significant relationship between serum FFA level and PPA in the trunk. Wave reflection from the upper body has little or no impact on the aortic pressure, whereas wave reflection from the lower body has a considerable impact on the aortic pressure.^(7)^ Despite the fact that wave reflection principally determines PPA,^(7)^ PPA in the arm does not evaluate the effective reflection site. This probably has led to the controversial relationship between FFA and PPA. However, PPA in the trunk evaluates a major reflecting site (i.e., femoral artery),^(8)^ and therefore, the limited wave reflection by the FFA-induced disappearance of arterial stiffness gradient may negatively (or less positively) affects PPA in the trunk. In line with this hypothesis, the regression model showed that circulating FFA has no significant influence on PPA in the trunk.

The longitudinal study confirmed that serum FFA level was significantly associated with PPA in the arm but not the trunk. The mean value of PPA in the arm did not change after the 12-week lifestyle intervention, however, there was a significant relationship between the individual changes in both PPA in the arm and serum FFA level. On the other hand, the mean value of PPA in the trunk significantly increased after the intervention, and there was no significant association between the individual changes in both PPA in the trunk and serum FFA level. No previous study had shown that lifestyle interventions significantly increased PPA irrespective of its evaluating points. However, we cannot conclude that lifestyle intervention can increase PPA in the trunk because this longitudinal study was a one-arm trial without a control group. Although obesity indices dramatically decreased after the intervention, serum FFA level increased significantly. There has been no consistent result on the effects of weight loss on circulating FFA level.^(25–27)^ The increase in serum FFA level seems to attribute to the negative energy balance (prolonged fasting period) and the suppressed insulin secretion; however, the exact mechanisms behind the elevated serum FFA level after the lifestyle intervention are unclear.

The major limitation of the current study was that the study population was limited to men aged 30–64 years to reduce the influences of age and sex on FFA and PPA; therefore, further investigations are needed to generalize the findings to other populations.

In conclusion, serum FFA level was associated with PPA in the arm but not trunk in overweight/obese men. The findings suggest that PPA in the trunk should be measured instead of PPA in the arm to solve the paradox (i.e., the favorable relationship between FFA and PPA) and to simplify the interpretation of PPA.

## Figures and Tables

**Table 1:** Subjects’ characteristics (*n* = 85) in the cross-sectional study

Age (years)	50 ± 9
Height (cm)	170.6 ± 6
Body mass (kg)	83.1 ± 9.1
Body mass index (kg/m^2^)	28.5 ± 2.4
Waist circumference (cm)	98.7 ± 6.8
HDL cholesterol (mmol/L)	1.35 ± 0.28
LDL cholesterol (mmol/L)	3.29 ± 0.85
Triglycerides (mmol/L)	1.73 ± 1.26
Free fatty acid (µmol/L)	531 ± 200
Fasting glucose (mmol/L)	5.41 ± 1.01
Fasting insulin (pmol/L)	71.3 ± 40.4
HOMA-IR (U)	2.6 ± 1.8
Aortic SBP (mmHg)	128 ± 15
Aortic PP (mmHg)	42 ± 8
Brachial SBP (mmHg)	134 ± 15
Brachial PP (mmHg)	48 ± 8
Femoral SBP (mmHg)	135 ± 15
Femoral PP (mmHg)	49 ± 9
Mean blood pressure (mmHg)	105 ± 12
Diastolic blood pressure (mmHg)	86 ± 10
Time to reflection (msec)	142 ± 15
Ejection duration (msec)	315 ± 24
Heart rate (bpm)	64 ± 10
PWV_carotid-femoral_ (cm/s)	884 ± 132
PWV_femoral-ankle_ (cm/s)	925 ± 98
PPA_aorta-femoral_ (%)	116 ± 10
PPA_aorta-brachial_ (%)	114 ± 8

Data are means ± SD. HDL, high-density lipoprotein; LDL, low-density lipoprotein; HOMA-IR, homeostasis model assessment for insulin resistance; SBP, systolic blood pressure; PP, pulse pressure; PWV, pulse wave velocity; PPA, pulse pressure amplification.

**Table 2:** Regression models for PPA_aorta-femoral_ and PPA_aorta-brachial_ as dependent variables in the cross-sectional study

Independent variables	PPA_aorta-femoral_			PPA_aorta-brachial_	
	β	*p*		β	*p*
Age	0.150	0.236		0.157	0.180
Height	0.188	0.191		0.051	0.701
Body mass*	–0.072	0.745		0.019	0.925
Waist circumference	–0.068	0.725		–0.221	0.218
HDL cholesterol*	–0.195	0.091		–0.045	0.671
LDL cholesterol*	–0.007	0.940		–**0.202**	**0.026**
Triglycerides*	–0.083	0.473		–0.101	0.346
Free fatty acid*	0.002	0.982		**0.199**	**0.036**
HOMA-IR*	0.014	0.903		0.187	0.092
Mean blood pressure	0.004	0.977		–0.087	0.463
Time to reflection	**0.376**	**0.003**		0.215	0.063
Ejection duration	–**0.755**	**<0.001**		–**0.688**	**<0.001**
PWV_carotid-__femoral_	–**0.304**	**0.040**		–**0.290**	**0.034**
PWV_femoral-__ankle_	0.112	0.399		–0.082	0.501
Currently smoking	0.016	0.859		–0.013	0.878
Medications for hypertension	0.083	0.402		0.130	0.161

Model for PPA_aorta-femoral_: *R*^2^ = 0.449, *p*<0.001. Model for PPA_aorta-brachial_: *R*^2^ = 0.502, *p*<0.001. *Log_10_-transformed. Numbers in bold indicate statistical significance. PPA, pulse pressure amplification; HDL, high-density lipoprotein; LDL, low-density lipoprotein; HOMA-IR, homeostasis model assessment model for insulin resistance; PWV, pulse wave velocity.

**Table 3:** Participants’ characteristics (*n* = 33) before and after the 12-week lifestyle intervention

	Before	After
Age (years)	51 ± 9	
Height (cm)	169.6 ± 5.2	
Body mass (kg)	82.1 ± 6.4	69.8 ± 5.8***
Body mass index (kg/m^2^)	28.5 ± 1.8	24.3 ± 1.7***
Waist circumference (cm)	97.9 ± 4.9	84.7 ± 5.3***
HDL cholesterol (mmol/L)	1.33 ± 0.24	1.42 ± 0.29*
LDL cholesterol (mmol/L)	3.15 ± 0.7	2.76 ± 0.70**
Triglycerides (mmol/L)	2 ± 1.73	0.84 ± 0.41***
Free fatty acid (µmol/L)	532 ± 172	669 ± 175***
Fasting glucose (mmol/L)	5.23 ± 0.79	4.8 ± 0.40**
Fasting insulin (pmol/L)	56.6 ± 28.1	30.3 ± 12.7***
HOMA-IR (U)	1.95 ± 1.01	0.95 ± 0.44***
Aortic SBP (mmHg)	128 ± 13	113 ± 8***
Aortic PP (mmHg)	43 ± 8	39 ± 5**
Brachial SBP (mmHg)	134 ± 13	117 ± 8***
Brachial PP (mmHg)	49 ± 8	43 ± 5***
Femoral SBP (mmHg)	134 ± 13	121 ± 9***
Femoral PP (mmHg)	49 ± 9	46 ± 6^†^
Mean blood pressure (mmHg)	104 ± 10	92 ± 8***
Diastolic blood pressure (mmHg)	85 ± 9	74 ± 8***
Time to reflection (msec)	141 ± 15	153 ± 13***
Ejection duration (msec)	317 ± 21	333 ± 18***
Heart rate (bpm)	63 ± 8	56 ± 7***
PWV_carotid-femoral_ (cm/s)	874 ± 124	809 ± 120***
PWV_femoral-ankle_ (cm/s)	914 ± 119	862 ± 80**
PPA_aorta-femoral_ (%)	115 ± 9	120 ± 8***
PPA_aorta-brachial_ (%)	114 ± 6	112 ± 6^‡^

Data are means ± SD. *p<0.05, **p<0.01, ***p<0.001, ^†^*p* = 0.06, ^‡^*p* = 0.09 vs before the intervention. HDL, high-density lipoprotein; LDL, low-density lipoprotein; HOMA-IR, homeostasis model assessment for insulin resistance; SBP, systolic blood pressure; PP, pulse pressure; PWV, pulse wave velocity; PPA, pulse pressure amplification.
